# Endogenous auxin and jasmonic acid levels are differentially modulated by abiotic stresses in rice

**DOI:** 10.3389/fpls.2013.00397

**Published:** 2013-10-09

**Authors:** Hao Du, Hongbo Liu, Lizhong Xiong

**Affiliations:** National Key Laboratory of Crop Genetic Improvement and National Center of Plant Gene Research (Wuhan), Huazhong Agricultural UniversityWuhan, China

**Keywords:** *Oryza sativa*, abiotic stress, auxin, jasmonic acid

## Abstract

Abiotic stresses such as drought, salinity, and adverse temperatures are major limiting factors for plant growth and reproduction. Plant responses to these stresses are coordinated by arrays of regulatory networks including the induction of endogenous abscisic acid (ABA), a well documented phytohormone for stress responses. However, whether or how these abiotic stresses affect the endogenous biosynthesis or metabolism of other phytohormones remains largely unknown. Here, we report the changes of endogenous indole-3-acetic acid (IAA) and jasmonic acid (JA) levels and expression of genes related to the biosynthesis or signaling of these hormones in rice under various abiotic stress conditions. The IAA content was decreased after drought stress, but it was significantly increased under cold and heat stresses. And the auxin-regulated gravitropism of root tip was inhibited by cold stress. Many genes involved in the IAA biosynthesis and signaling were changed in transcript level under these stresses, and the changes were essentially in agreement with the change of endogenous IAA level. Interestingly, the endogenous JA content was increased markedly under drought and cold stresses, but it was reduced by heat stress. Accordingly, many genes involved in JA biosynthesis and signaling were induced by drought and cold treatment but these genes were significantly suppressed by heat stress. We concluded that endogenous levels of IAA and JA were differentially regulated by abiotic stresses in rice, implying diverse roles of these hormones in stress responses.

## Introduction

Plant responses to abiotic stresses are coordinated by arrays of growth and developmental programs, which involves a variety of biochemical and physiological mechanisms that allow them to adapt to adverse conditions throughout the whole life cycle (Cushman and Bohnert, 2000). In most cases, plants respond to environmental stresses by changing the levels of endogenous phytohormones. For example, endogenous ABA level in rice was dramatically increased (10–50-fold) under drought stress (Du et al., 2010). ABA is well known for its important roles in facilitating the adaptation processes during drought and cold stresses (Xiong et al., 2002). Other phytohormones such as indole-3-acetic acid (IAA) and jasmonic acid (JA) have also been suggested to be involved in responses to abiotic stresses in Arabidopsis (Wang et al., 2001). However, little is known about the changes of endogenous levels of the two phytohormones in response to abiotic stresses in cereal crops.

IAA (or auxin) is a phytohormone well-known for its essential roles in plant morphogenesis, including tropistic growth, root patterning, vascular tissue differentiation, auxiliary bud formation, and flower organ development (Zhao, 2010). Cold stress and auxin contents are potentially linked since cold stress inhibits the root gravity response in Arabidopsis (Fukaki et al., 1996; Wyatt et al., 2002). Recent report suggested that the local auxin concentration and auxin distribution may be regulated by changes in auxin transport in plants under cold stress (Shibasaki et al., 2009). It was also reported that osmotic stress caused by increased salinity or drought had an impact on polar auxin transport (Wang et al., 2009b). In Arabidopsis, activation of the *YUCCA6* (*YUC6*) gene, encoding a flavin monooxygenase and functioning in the tryptophan-dependent auxin biosynthetic pathway, resulted in elevated endogenous auxin levels and enhanced drought resistance (Kim et al., 2013). Recent reports imply that the cold-induced changes in plant growth and development are likely linked to the intracellular auxin gradient (Shibasaki et al., 2009). In Arabidopsis, the auxin signaling mutants *axr1* and *tir1*, which showed reduced gravity response, responded to cold treatment similar to the wild-type, suggesting that cold stress may not affect auxin signaling (Shibasaki et al., 2009). Additionally, PIN3, an auxin transporter that has been suggested to mediate the early phase of the root gravity response, was inhibited by cold stress (Shibasaki et al., 2009), suggesting that cold stress may affect auxin transport. In rice, transcript profiling analysis revealed that many auxin-responsive genes are also responsive to cold stress (Jain and Khurana, 2009). Furthermore, our study demonstrated that the *OsGH3-2* overexpression rice decreased free IAA content, and showed increased resistance to cold stress due to the combined effects of IAA and ABA (Du et al., 2012). Most recently, we found that ABA or carotenoid-deficient mutants had reduced IAA content and exhibited increased cold resistance (Du et al., 2013).

JA is also an important plant developmental regulator involved in callus growth, seed germination, primary root growth, flowering, fertilization, and senescence (Feussner and Wasternack, 2002). This hormone is also involved in plant responses to insect wounding, infection of various pathogens, and various abiotic stresses (Pauwels et al., 2009). Pathogen attack stimulated the biosynthesis of endogenous JA, and exogenous application of JA to plants activated the expression of stress-related or pathogenesis-related (PR) genes (Moons et al., 1997; Mei et al., 2006). Compared to the massive studies on the role of JA in the response to biotic stresses, relatively less has been known about its role under abiotic stresses. Previous studies showed that both drought and high salinity caused increased JA levels in the leaves and roots of rice (Moons et al., 1997; Kiribuchi et al., 2005). Transgenic rice overexpressing Arabidopsis JA carboxyl methyltransferase gene (*AtJMT*) showed increased level of methyl jasmonate (MeJA) in young panicles and inhibition of spikelet development under drought condition, indicating that plants can produce MeJA during drought stress, which in turn may stimulate the production of ABA, together leading to a loss of grain yield (Kim et al., 2009). Recently, transgenic rice overexpressing *OsbHLH148*, a member of bHLH family gene in rice, showed increased tolerance to drought stress, and OsbHLH148 was proposed to act in the upstream signaling pathway of JA by forming an OsbHLH148-OsJAZ1-OsCOI1 signaling module (Seo et al., 2011), indicating JA signal pathway involved in drought response.

Although the change of JA or IAA under certain abiotic stress conditions had been documented, the changes of the two hormones have not been compared in the same background under the same conditions. Here, we compared the changes of the two hormones in rice under abiotic stresses. In addition, we also examined the expression levels of all known or predicted genes functioning in biosynthesis and signaling pathway of IAA and JA in rice under the stress treatments. The results from this study will help us understanding the different roles of the two hormones under abiotic stresses.

## Results

### Abiotic stresses modulate IAA and JA homeostasis in rice

**Figure 1:**
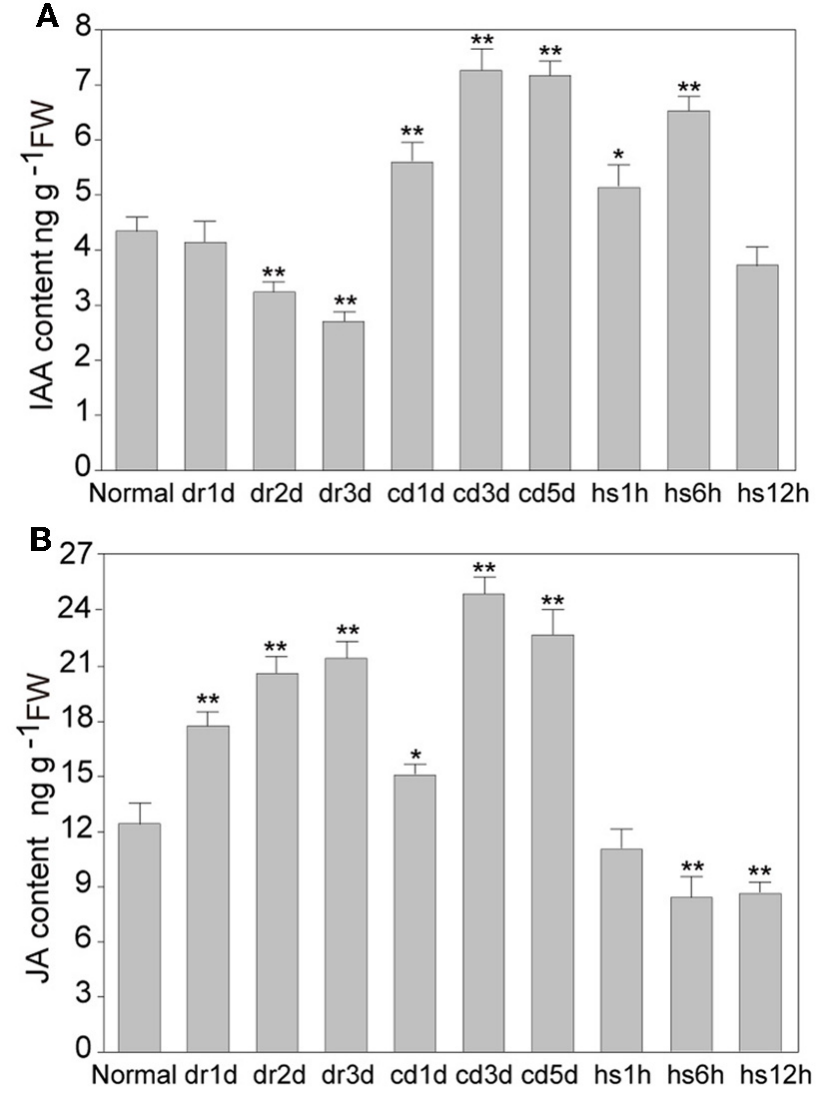
**Quantification of IAA and JA contents in rice**. **(A)** Quantification of IAA content in the leaves of rice seedling under normal and stress conditions. **(B)** Quantification of JA content in the leaves of rice seedling under normal and stress conditions. DR, drought; CD, cold; HS, heat stress; d, day; h, hour. ^*^ and ^**^ indicate significance (*t*-test) at *P* < 0.05 and *P* < 0.01 level, respectively. Values are means ± SD (*n* = 3).

To investigate the influence of abiotic stresses on IAA and JA, we examined the level of the two hormones in rice under different abiotic stresses. After slight drought stress (day 1 when some leaves slightly rolled), the IAA level had no significant change; after moderate drought stress (day 2 when some leaves completely rolled), IAA level was reduced to about 81% of the control; and after severe drought stress (day 3 when all leaves became rolled), the IAA level was reduced to 72% of the control (Figure 1). However, after cold stress for one day, the IAA level was increased to 1.2-fold of the control, and it was increased to 1.6-fold on the third day after cold stress (Figure 1). After heat stress for 1 h, the IAA level was slightly increased (1.1-fold), and it was increased to about 1.3-fold after 6 h of heat stress (Figure 1). Interestingly, the IAA level was reduced to the normal level after heat stress for 12 h (Figure 1). The JA content was also measured in the same leaf samples from stress-treated rice plants. JA level was significantly increased, reaching to 1.5-fold compared to the control after severe drought stress (Figure 1). After cold stress, JA level was induced markedly to about 2-fold compared to the control (Figure 1). Under heat stress for 1 h, no significant difference of JA level was detected. However, after heat stress for 6 or 12 h, the JA level was reduced to about 85% of the control. The results indicated that abiotic stresses, such as drought, cold and heat, differentially modulate the endogenous levels of IAA and JA.

### Expression profiling analysis of IAA biosynthesis or metabolism-related genes under abiotic stress

**Figure 2:**
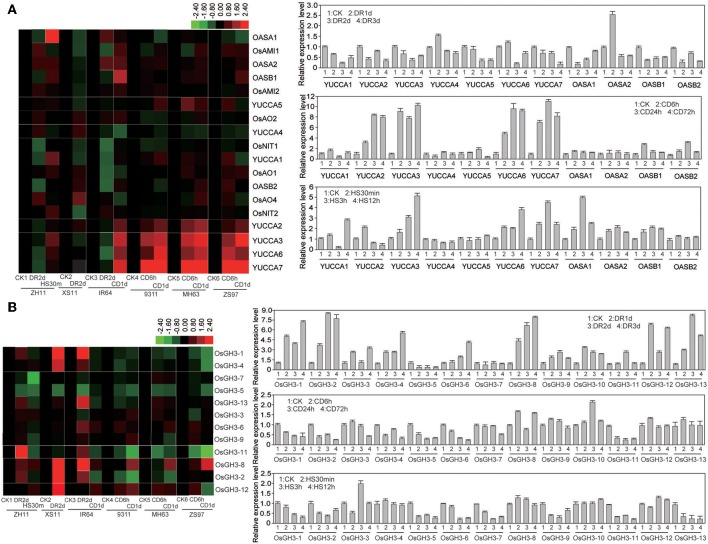
**Expression levels of IAA metabolism-related genes**. **(A)** Microarray and quantitative PCR analyses of auxin biosynthesis-related genes under normal or abiotic stress conditions. **(B)** Microarray and quantitative PCR analyses of auxin metabolism-related genes under normal or abiotic stress conditions. Two *japonica* rice Zhonghua 11 (ZH11) and Xiushui 11 (XS11), and four *indica* rice Zhenshan (ZS97), Minghui 63 (MH63), 9311, and IR64 were used for DNA chips (Note: varieties were referenced for different stress). ZH11 was used for quantitative PCR analysis. CK, control; DR, drought; CD, cold; HS, heat stress.

Since abiotic stresses affected endogenous level of IAA, we examined the transcript levels of the auxin metabolism related genes in several rice varieties (see Materials and Methods) after drought, cold, or heat treatments at seedling stage by Affymetrix microarray and quantitative PCR (qPCR) techniques. After statistical analysis of the microarray data, we found that many auxin biosynthesis-related genes including anthranilate synthase (AS) gene encoding a key enzyme in the synthesis of tryptophan (Trp), IAA, and indole alkaloids. In rice, *OsASA1*/*OsASA2* and *OsASB1*/*OsASB2* encode AS alpha and beta subunit, respectively (Tozawa et al., 2001). These genes were significantly suppressed under drought stress (Figure 2A). *OsOASB1* and *OsOASB2* were slightly induced by cold stress, but *OsOASA1* and *OsOASA2* were up-regulated by heat stress (Figure 2A). It is known that IPA can be further converted by YUCCA (flavin monooxygenase) to produce IAA. Of the seven YUCCA family genes in rice, six genes (except for *OsYUCCA4*) were down-regulated under drought stress (Figure 2A). However, the transcript levels of *OsYUCCA2, OsYUCCA3, OsYUCCA6*, and *OsYUCCA7* were strongly induced up to 10-fold by the cold stress (Figure 2A). Under the heat stress, *OsYUCCA3, OsYUCCA6*, and *OsYUCCA7* were quickly induced (about 5-fold) (Figure 2A). The maintenance of IAA homeostasis is also contributed by the conversion of active IAA to an inactive form via conjugation of IAA with amino acids (such as Asp, Ala, and Phe), and this conversion is catalyzed by IAA-amido synthetases belonging to the GH3 family (Staswick et al., 2005). Of the 13 GH3 members in rice genome, *OsGH3-1, OsGH3-2, OsGH3-8, OsGH3-12*, and *OsGH3-13* were markedly induced by the drought stress (Figure 2B). However, under cold stress, *OsGH3-1, OsGH3-2, OsGH3-5, OsGH3-6*, and *OsGH3-11* were down-regulated (Figure 2B). Furthermore, *OsGH3-1, OsGH3-2, OsGH3-5, OsGH3-6, OsGH3-7, OsGH3-9, OsGH3-11*, and *OsGH3-13* were reduced significantly by heat stress (Figure 2B). These results suggested that the IAA biosynthesis genes were mainly up-regulated by cold and heat stresses, but suppressed by drought stress. In addition, the expression changes of IAA catabolism-related genes such as *GH3* family were contrary to the changes of IAA biosynthesis genes under the abiotic stresses. These results further supported the different changes of IAA level under different stresses. In general, the change patterns of these genes were consistent among the different rice varieties compared, but some genes, such as *OASA1* and *YUCCA4* under drought condition, and *OASB2, YUCCA1*, and *YUCCA5* under cold condition, showed slight difference among the rice varieties, indicating a natural variation in the modulation of endogenous IAA levels at gene expression level.

### Expression levels of IAA signaling and polar transport related genes under abiotic stresses

**Figure 3:**
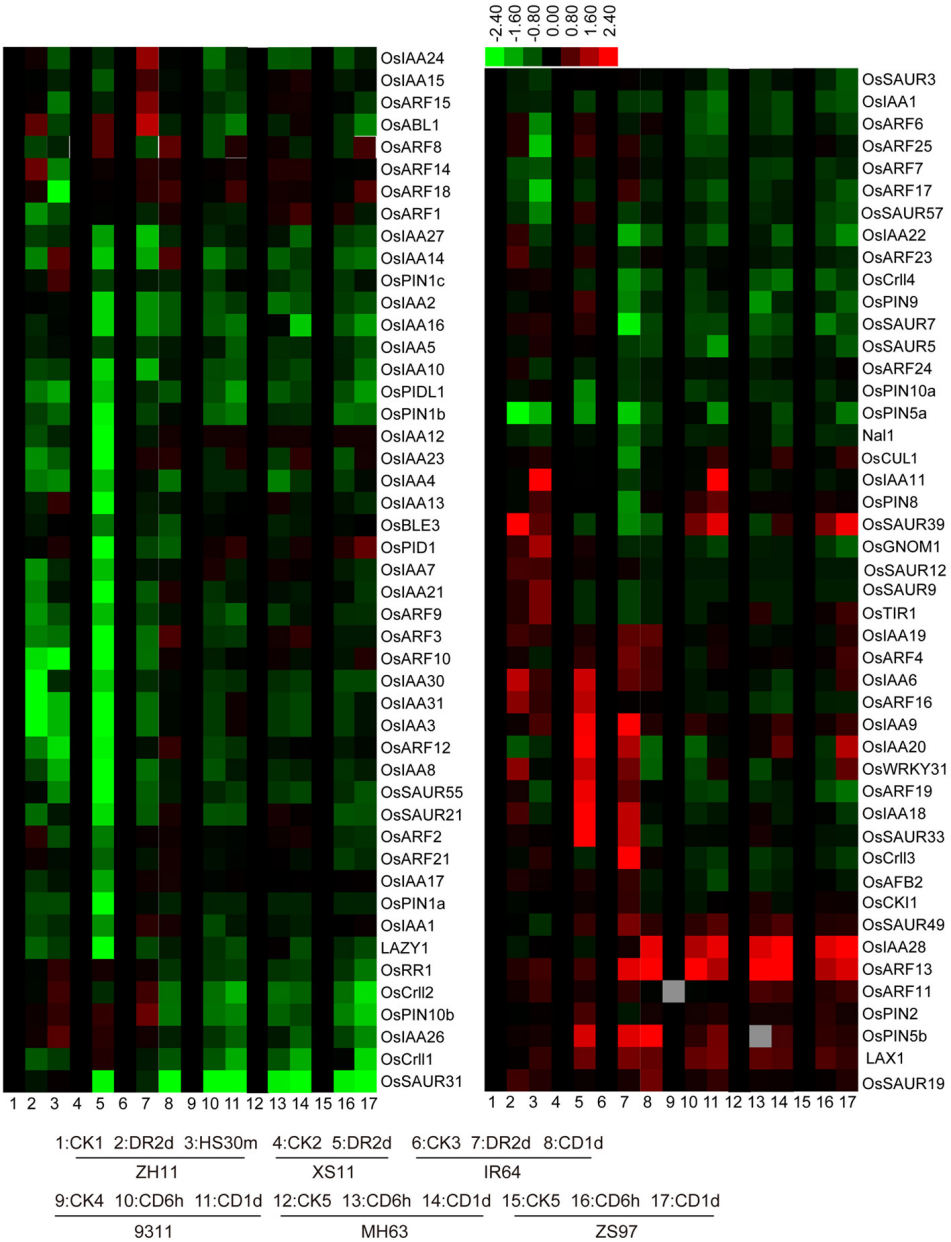
**Expression levels of IAA signaling-related genes**. The rice variety names are the same as indicated in Figure 2. CK, control; DR, drought; CD, cold; HS, heat stress.

In general, IAA homeostasis is also related to IAA polar transport and signaling pathway. Therefore, we further examined the reported IAA signaling and polar transport-related genes. Aux/IAA proteins and auxin response factors (ARFs) have been well recognized for their roles in auxin signaling. Aux/IAA proteins are short-lived transcriptional regulators that mediate auxin responses through interaction with ARFs (Reed, 2001). Among the 31 *OsIAA* and 25 *OsARF* genes, most were suppressed by drought, cold, and/or heat stresses, while some of the *OsIAA* genes (*OsIAA6, OsIAA9, OsIAA18, OsIAA19, OsIAA20*, and *OsIAA28*) and *OsARF* genes (*OsARF4, OsARF11, OsARF13, OsARF14, OsARF16, OsARF18*, and *OsARF19*) were induced by at least one of the stresses (Figure 3). It has been reported that the ubiquitin-ligase complex (Skp1-CUL1-F-box[SCF])^TIR1^ acts as an auxin receptor in auxin signaling (Tan et al., 2007). In rice, *OsAFB2, OsTIR1*, and *OsCUL1* are putative orthologs in the complex of auxin signal reception (Xia et al., 2012). The transcript level of *OsAFB2* was reduced under cold stress, but the transcript level of *OsTIR1* was increased under drought and heat stresses, and it was decreased under cold stress (Figure 3). Nevertheless, the expression of *OsCUL1* had no obvious change under these stresses (Figure 3). *Crl1* (*Crown rootless1*) was characterized as a positive regulator for crown and lateral root formation and its expression was directly regulated by an ARF in auxin signaling pathway (Inukai et al., 2005). There are four *Crl1* homologs in rice genome, and their expressions were suppressed by drought, heat, or cold stresses (Figure 3). Small auxin-up RNAs (SAURs) are early auxin-responsive genes existing in plants as a large gene family (Franco et al., 1990), and they act as negative regulators of auxin synthesis and transport in rice (Kant et al., 2009). Nine *OsSAUR* genes (*OsSAUR3, OsSAUR5, OsSAUR7, OsSAUR9, OsSAUR12, OsSAUR21, OsSAUR31, OsSAUR55*, and *OsSAUR57*) were significantly decreased under cold stress, but *OsSAUR39* and *OsSAUR49* were induced by cold stress (Figure 3). *LAX1*, encoding a bHLH transcription factor in rice and functioning in the initiation of auxiliary meristem formation by altering auxin response or transport (Oikawa and Kyozuka, 2009), was induced by all three treatments (Figure 3). However, *LAZY1*, a novel grass-specific gene that controls rice shoot gravitropism through negatively regulating polar auxin transport (Li et al., 2007), was suppressed by the treatments (Figure 3). PIN-FORMED (PIN) proteins are secondary transporters acting in the efflux of auxin from cells (Blakeslee et al., 2005). There are 12 *OsPINs* in rice genome (Wang et al., 2009a) but only 10 *OsPINs* were detected in the microarray. *OsPIN2* and *OsPIN5b* were induced by drought, heat, and cold stresses while the others were significantly suppressed by the stresses (Figure 3). PINOID (PID), a serine threonine protein kinase in *Arabidopsis*, was reported for its role in auxin distribution through a positive control of subcellular localization of PINs (Robert and Offringa, 2008). Among the PID-like genes detected in the microarray analysis, *OsPID1* and *OsPIDL1* were suppressed by drought, heat, and/or cold stresses (Figure 3). These results together suggested that abiotic stresses also affected the expressions of IAA signaling and polar transport-related genes.

### Expression levels of JA metabolism ABD signaling-related genes under abiotic stress

**Figure 4:**
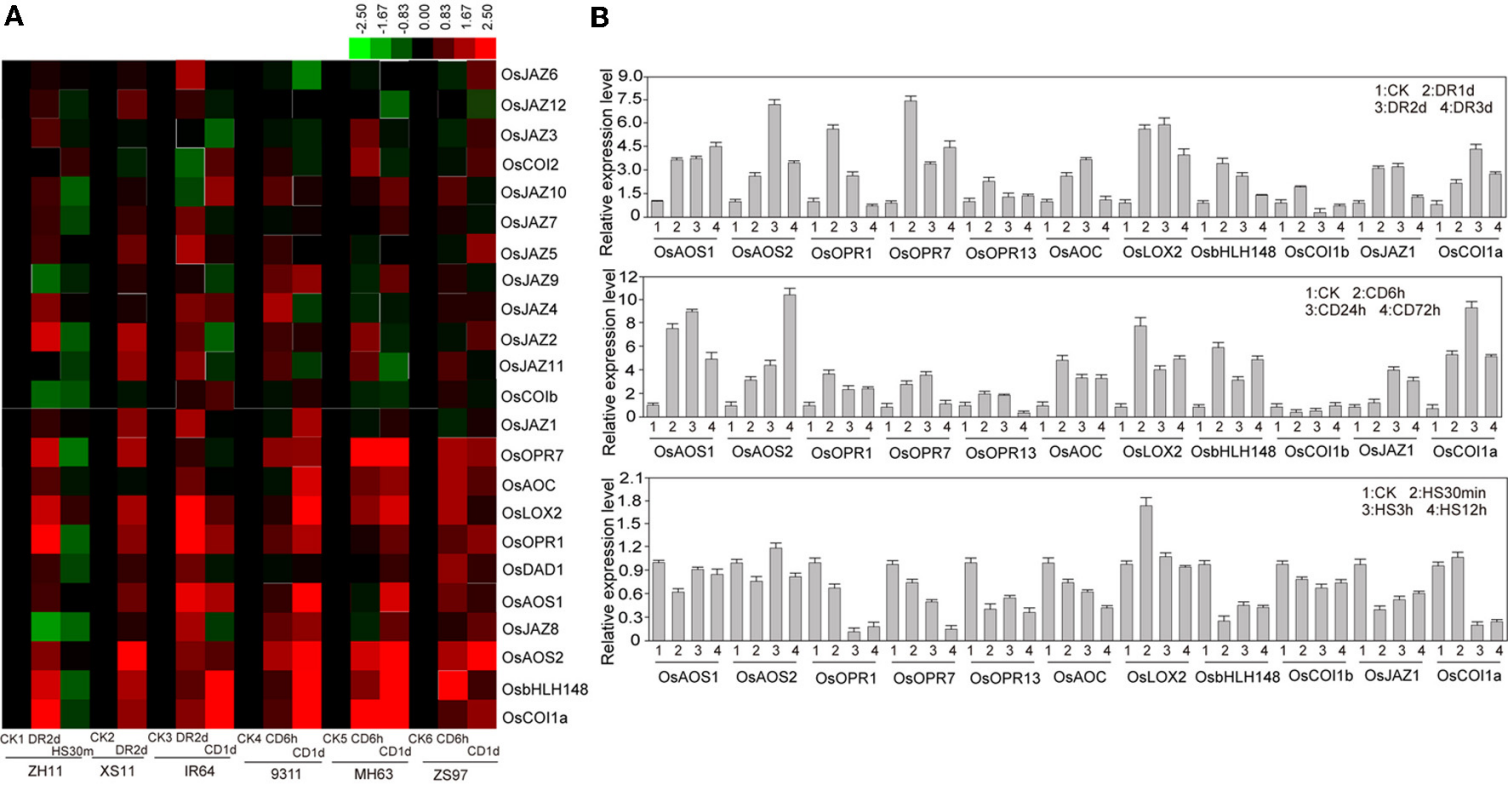
**Expression levels of JA related genes**. **(A)** Microarray analysis of JA biosynthesis or signal related genes under normal or abiotic stress conditions. The rice variety names for microarray analysis are the same as indicated in Figure 2. **(B)** Quantitative PCR analysis of JA biosynthesis or signal related genes in rice ZH11 under normal or abiotic stress conditions. CK, control; DR, drought; CD, cold; HS, heat stress.

In rice genome, the reported genes participated in the biosynthesis of JA include *OsDAD1, OsLOX2, OsAOC, OsAOS1, OsAOS2, OsOPR1*, and *OsOPR7*. These genes were induced by drought and cold stresses, but down-regulated by heat stress (Figure 4). JA can be conjugated with isoleucine by JARs that belong to GH3 family encoding IAA-amino synthetases. Reported examples of these genes in rice including *OsJAR1/OsGH3-5, OsJAR2/OsGH3-3*, and the expression of *OsGH3-5* was decreased significantly under drought, heat, and cold stresses, however, expression of *OsGH3-3* was not significantly changed under these stresses (Figure 4). Pervious study showed that the SCF^COI1^ E3 ubiquitin ligase complex acted as JA receptor (Yan et al., 2009). There are three COI1 homologs (*OsCOI1a, OsCOI1b*, and *OsCOI2*) in rice. *OsCOI1a* was reported to form an SCF complex and to regulate the expression of OsbHLH148 upon coronatine treatment (Seo et al., 2011). According to the microarray data, both *OsbHLH148* and *OsCOI1a* were up-regulated significantly after drought and cold stresses, but they were decreased markedly under heat stress (Figure 4). *OsCOI1b* was suppressed by drought; however, *OsJAZ1* was induced by drought and cold stresses according to the microarray and qPCR results (Figure 4). JAZ proteins are also involved in JA signaling. There are 12 OsJAZ members in rice genome. Most of the *OsJAZ* genes were strongly induced by drought stress but were suppressed by heat stress (Figure 4). These results together suggest that JA metabolism and signaling pathways are significantly regulated by the abiotic stresses.

### Effect of cold stress on rice root gravity response

**Figure 5:**
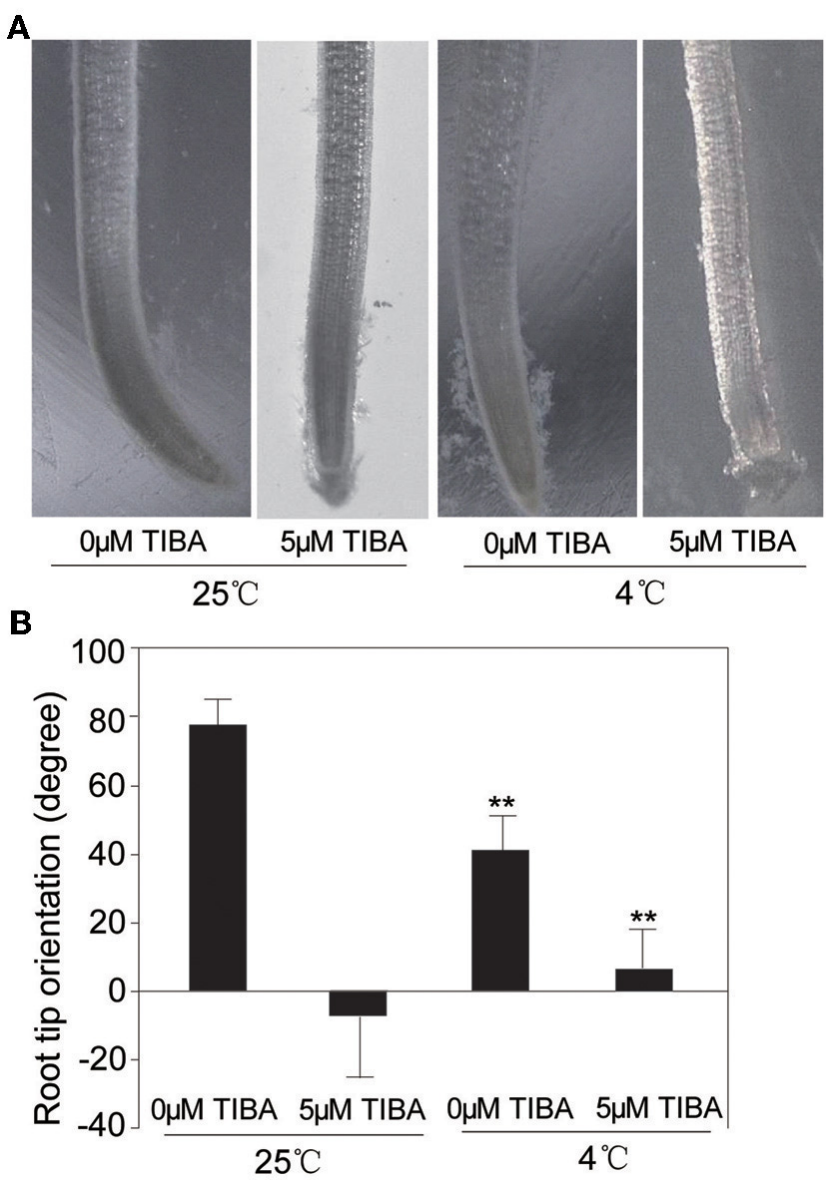
**Performance of the orientation of rice root tip under gravity**. **(A)** Morphological phenotypes of the orientation of root tip by gravity under normal or cold (4°C) conditions with or without 5 μM TIBA. **(B)** Statistical analysis the angle of horizontal orientation of root tip. ^**^ indicates significance (*t*-test) at *P* < 0.01 level. Values are means ± SD (*n* = 3).

IAA level was increased under cold stress, which prompted us to investigate if the IAA-related gravity response of rice had any change under cold condition. Rice seedlings were planted in MS medium for 3 days and then were vertically oriented for growth at 4°C and 25°C, respectively. To further elucidate the effect of cold stress on gravity response of rice root, 5 μM TIBA (2,3,5-triiodobenzoic acid), an auxin transport inhibitor, was added to MS medium, and the root bending was measured at the second day after treatment. The result showed that, under the normal conditions, the oriented angle was about 78°. However, the TIBA treatment almost completely inhibited the gravitropism of rice root, with the root tip orientation angle about 0° (Figure 5). Under the cold stress without TIBA treatment, the orientation angle was about 45°, suggesting that cold stress significantly inhibited the gravitropism of root tips. Upon treatments of 5 μM TIBA and cold stress, the orientation angle was about 8°, significantly different to the root orientation under the normal conditions (Figure 5). These results indicated that gravity response of root tips was inhibited by cold stress, which may due to the reduced IAA level under cold stress.

## Discussion

### The biosynthesis of IAA was INVOVED in abiotic stress

IAA plays important roles in plant development and also in responses to abiotic stresses. IAA biosynthesis has been intensively studied in Arabidopsis. Several groups found a two-step conversion of Trp to IAA. In this conversion, Trp is first converted to IPA by amino transferases belonging to TAA family, and IAA is produced from IPA by the YUCCAs (Mashiguchi et al., 2011; Won et al., 2011). YUCCAs are flavin monooxygenases catalyzing the NADPH-dependent hydroxylation of IPA, which is a rate-limiting step in tryptophan-dependent IAA biosynthesis (Zhao et al., 2001; Zhao, 2012). The maintenance of IAA is also contributed by the conversion of active IAA to inactive form via conjugation of IAA with amino acids (such as Asp, Ala, and Phe) by IAA-amido synthetases belonging to the GH3 family. The GH3 proteins are conserved in monocots and dicots, and there are 13 members in rice (Staswick et al., 2005; Jain et al., 2006). Plants subjected to oxidative stress exhibited various phenotypic changes that are related to alterations in auxin level and distribution (Pasternak et al., 2005). In our recent reports, transgenic rice overexpressing *OsGH3-2* showed decreased free IAA content and different alterations in drought and cold tolerance (Du et al., 2012); and the carotenoid-deficient rice mutants with decreased IAA level showed increased resistance to cold stress (Du et al., 2013). These reported results suggest that the homeostasis of auxin level is closely related to cold and drought tolerance. In this study, a few AS family genes were altered under drought, cold, and heat stresses, and many *YUCCA* genes were suppressed by drought stress, but some of *YUCCA* genes were strongly induced by cold and heat stresses (Figure 2), indicating that auxin biosynthesis may be activated by cold or heat stress, but suppressed by drought stress. IAA-amino synthesizes of GH3 family were reported for their negative roles in controlling endogenous IAA level in plants more than two decades ago (Franco et al., 1990). *OsGH3-13*-overexpression rice showed enhanced drought tolerance and reduced free IAA level (Zhang et al., 2009), also demonstrating the importance of auxin in stress tolerance. Among the OsGH3 family, five members (*OsGH3-1, OsGH3-2, OsGH3-8, OsGH3-12*, and *OsGH3-13*) were markedly induced by drought stress. However, five members (*OsGH3-1, OsGH3-2, OsGH3-5, OsGH3-6*, and *OsGH3-11*) were down-regulated by cold stress (Figure 2B). Many *OsGH3* genes (*OsGH3-2, OsGH3-5, OsGH3-6, OsGH3-7, OsGH3-9, OsGH3-11*, and *OsGH3-13*) were down-regulated by heat stress (Figure 2B). These expression data are generally in agreement with the different changes of endogenous IAA level under drought, cold, and heat stresses (Figure 1A). Our results suggest that IAA has distinct roles in the responses of rice to different stresses. Through the regulation of endogenous auxin biosynthesis, plants may establish a new accommodative status for adaptation to the adverse environmental cues.

### IAA signaling and polar transport may be affected by abiotic stress

Although a line of reports provide some clues on the involvement of auxin signaling in stress responses (Hannah et al., 2005; Song et al., 2009), the exact mechanism of auxin-mediated stress responses remains to be elucidated. Recently, ABI5-Like1 (ABL1), a rice homolog of the ABA signaling component ABI5, was proposed for its possible role in modulating auxin responses by directly regulating the expression of ABRE-containing genes related to auxin metabolism or signaling (Yang et al., 2011). Many auxin-signaling genes are responsive to stress responses. In a previous study, the expression profiles of all rice Aux/IAA genes in different tissues and under abiotic stresses were examined by qPCR analysis, and some members showed specific expression while some genes had overlapping expression patterns (Jain and Khurana, 2009; Song et al., 2009). In this study, we found most *OsIAA* and *OsARF* genes were differentially responsive to drought, heat and cold stresses (Figure 3), indicating an interaction between plant growth and abiotic stress. *OsAFB2, OsTIR1*, and *OsCUL1* described as putative auxin receptor associated genes (Xia et al., 2012), and these genes were differentially regulated by drought, cold, and heat stresses (Figure 3), suggesting that the upstream of auxin signaling process may be also influenced by these stresses. The different responses of from the *OsSAUR* family genes under the stress treatments (Figure 3) also supported that auxin-signaling may be differentially regulated by abiotic stresses.

Abiotic stresses may also affect polar auxin transport, which is supported by a few studies reporting the altered expression of *PIN* genes (Blakeslee et al., 2005) and inhibition of polar auxin transport by phenolic compounds accumulated in response to stress exposure (Potters et al., 2009). In this study, two *OsPIN* genes (*OsPIN2* and *OsPIN5b*) were induced by drought, heat and cold stresses, however, the other members in rice were suppressed significantly by abiotic stress (Figure 3). PID proteins control auxin distribution through a positive control of subcellular localization of PIN (Robert and Offringa, 2008). Two PID-like gene in rice were suppressed by drought, heat, and cold stresses (Figure 3), indicating that auxin distribution may be also affected by abiotic stresses. In addition, the experiment of Arabidopsis root growth and gravity in response to stress suggested that cold affected the auxin response and the gravity response was regulated partially by the diffidence in auxin distribution and basipetal movement (Rashotte et al., 2000). Our result also showed that cold stress inhibited the gravitropism response of rice root tips (Figure 4).

### Abiotic stress affects JA biosynthesis and signaling pathway

The phytohormone jasmonate and its metabolites regulate plant growth and development processes and responses to environmental stimuli (Turner et al., 2002; Pauwels et al., 2009). Jasmonates, as well as octadecanoids, which comprise *cis*-(+)-12-oxophytodienoic acid (OPDA) and its metabolites, originate from α-linolenic acid (α-LeA) of chloroplast membranes. Upon oxygenation by 13-LIPOXYGENASE (13-LOX), an unstable allene oxide is formed by a 13-ALLENE OXIDE SYNTHASE (13-AOS) and subsequently cyclized by an ALLENE OXIDE CYCLASE (AOC) to *cis*-(+)-OPDA (Feussner and Wasternack, 2002; Wasternack, 2007; Zerbe et al., 2007). The levels of endogenous jasmonates were reported to be increased upon pathogen infection (Thomma et al., 1998). However, little is known about JA in response to abiotic stresses. Previous studies showed that jasmonate levels were increased upon exposure to drought and salt stresses (Creelman and Mullet, 1995; Wang et al., 2001). In rice, both drought and high salinity stresses resulted in increase of jasmonate levels in the leaves and roots and induction of JA biosynthesis genes (Moons et al., 1997; Tani et al., 2008). In this work, we found that the orthologs of JA biosynthesis genes in rice, including *OsDAD1, OsLOX2, OsAOC, OsAOS1, OsAOS2, OsOPR1*, and *OsOPR7* were remarkably up-regulated by drought stress (Figure 4), and this result agreed with the increased JA level upon drought. In addition, some genes with putative functions in JA signaling, such as *OsJAR1, OsbHLH148*, and *OsCOI1a*, were also differentially regulated by drought, cold stress, and heat stresses (Figure 4). Previous study showed that overexpression of *OsJAZ6* in rice resulted in improved tolerance to salt and mannitol stresses (Ye et al., 2009), indicating that JA signaling is also involved in abiotic stress responses in rice. *Another* recent study suggested that salt stress response may be modulated by a jasmonate signaling (Ismail et al., 2012). These results together suggest that JA biosynthesis and signaling differentially regulate the responses and adaptation of plants to diverse abiotic stresses.

**Figure 6:**
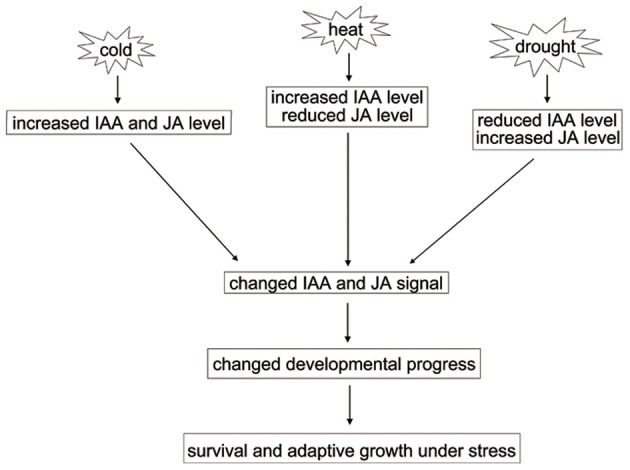
**A model for the effect of JA and IAA changes under abiotic stresses**.

Drought, cold, and heat stresses often cause different changes at physiological and molecular levels in plants (Yamaguchi-Shinozaki and Shinozaki, 2006). Our finding suggests that the biosynthesis and signaling of JA and IAA are differentially regulated by different abiotic stresses. We propose that the balance of JA and IAA homeostasis and signaling are critical for plant development and stress responses (a schematic model is shown in Figure 6). Further, investigation of the molecular mechanisms in modulating the balance of endogenous hormones will help elucidate the basic development programs and adaptive capacity of plants by integrating the signaling pathways of endogenous hormones and exogenous environmental stimuli.

## Materials and methods

### Plant materials and growth condition

The materials used for DNA microarray analysis included *japonica* rice Zhonghua 11 (*Oryza sativa* subsp. *japonica*) and Xiushui 11 (XS11) and *indica* rice Zhenshan 97, (ZS97), Minghui 63 (MH63), 9311, and IR64. ZH11 was used for qPCR, physiological and phenotypic analysis. To examine the transcript levels of genes under various stresses, Seeds of ZH11 plants were germinated on Murashige and Skoog (MS) medium in a growth chamber with 14 h light/10 h dark cycle for 4 days, and then the seedlings were transplanted into pots, for cold stress rice at the five-leaf stage were transferred to a growth chamber at 4°C and sampled at 0, 6, and 12 h after the treatment, and at 42°C for heat stress and sampled at 0 min, 30 min, 3 h and 12 h after the treatment. For drought stress, watering was stopped and leaves were sampled at the following time points: control, no stress; day 1 (when seedlings showed slight leaf rolling); day 2 (the second day after time point day 1); day 3 (the third day after time point day 1).

### Root bending assay

The seeds were sterilized with HgCl2 (0.15%) and germinated and grown in transparent plastic boxes with 1/2 MS medium (0.6% agar) in a growth chamber at 25°C or 4°C with a 14 h light/10 h dark cycle. After the seminal root reached a length of 2–3 cm, boxes were laid down, allowing plants to be turned at an angle of 90° to the horizontal plane, and grown for 1–2 days before measurement.

### Microarray analysis

An Affymetrix DNA chip containing all putative genes of the rice genome was used for investigating expression profile changes. RNA extracted from leaves of rice under normal and abiotic stress conditions were used for microarray analysis. Chip hybridization and data processing were carried out with Affymetrix custom service (CapitoBio, China) following the standard protocol. The microarray dataset of this research in supplemental Table S1.

### RNA extraction and qPCR

Rice RNA was isolated using Trizol reagent (*Invitrogen*, USA), for real time PCR, 5 μg total RNA was digested using DNase I and reverse-transcribed using RNase-free Superscript III reverse transcriptase (*Invitrogen*, USA) according to the manufacturer's instructions. The qPCR was done using SYBR Premix Ex Taq reagent (TaKaRa, Japan) with an ABI 7500 qPCR system (Applied Biosystems, USA). The details of the qPCR procedure were described previously (Du et al., 2010). An Rn threshold of 0.2 was used to obtain CT (threshold cycle) values for all of the amplification plots. The relative expression levels for all of the target genes were determined based on the 2^ΔΔ*CT*^ method (Livak and Schmittgen, 2001) using rice Actin1 as an internal control. The primers for qPCR are listed in Supplemental Table S2.

### Quantification of IAA and JA

To quantify IAA and JA contents, samples were ground to fine powder. For IAA and JA extraction, 100 mg of five-leaf-stage seedlings were extracted twice with 900 μL of extraction buffer [methanol:H_2_O:acetonitrile = 90:9:1(v/v)]. Quantification was performed in an ABI 4000Q-TRAR LC-MS system (Applied Biosystems, USA) with stable-isotope-labeled ABA and auxin as standards (OlChemIm, Czech Specials) according to a method described previously with miner modification (Liu et al., 2012).

### Conflict of interest statement

The authors declare that the research was conducted in the absence of any commercial or financial relationships that could be construed as a potential conflict of interest.

## Supplementary material

Table S1 **The microarray dataset used in this study**.

Table S2 **The primers for qPCR used in this study**.
